# Metabolic and Inflammatory Biomarkers, Multimorbidity Combinations and Disability Burden Among Older Americans

**DOI:** 10.1093/geroni/igaa057.713

**Published:** 2020-12-16

**Authors:** Anda Botoseneanu, Sheila Markwardt, Ana Quinones

**Affiliations:** 1 University of Michigan, Dearborn, Michigan, United States; 2 School of Public Health - Portland State University, Portland, Oregon, United States; 3 Oregon Health & Science University, Portland, Oregon, United States

## Abstract

Specific multimorbidity combinations, in particular those including arthritis, stroke, or cognitive impairment have been associated with high burden of ADL/IADL disability. However, the biologic underpinnings of these associations are still unclear. We used data from the Health & Retirement Study (N=5,359, age 65 years or older at baseline) and negative binomial regression models to evaluate if metabolic and inflammatory biomarkers [HbA1c, HDL-cholesterol, C-Reactive Protein (CRP)] mediate the association between specific multimorbidity combinations (at baseline in 2010-2012; grouped around one of eight index diseases: arthritis, cancer, cognitive impairment, diabetes, heart disease, hypertension, lung disease, and stroke) and ADL/IADL disability (at subsequent wave in 2012-2014). Results were adjusted for sociodemographic characteristics, body-mass index, number of coexisting chronic diseases, and baseline ADL/IADL score. HbA1c (IRR=1.01, p=0.004) and CRP (IRR=1.01, p=0.003), but not HDL, were positively associated with the number of coexisting diseases. After adjustment for coexisting diseases, higher HbA1c was associated with greater ADL/IADL limitations for multimorbidity combinations including arthritis (IRR=1.11, p=0.047) and stroke (IRR=1.11, p=0.047), but not for combinations centered around other diseases, while CRP was no longer significantly associated with ADL/IADL limitations for any of the multimorbidity combinations. Models accounting for HbA1c and CRP, respectively, showed that only combinations including cognitive impairment had greater ADL/IADL limitations (IRR=1.45, p=0.015) compared to combinations without cognitive impairment. Insulin resistance and inflammation are strongly associated with the burden of multimorbidity; this strong association, rather than the biomarkers per se, appears to explain the greater ADL/IADL burden observed with all disease-specific multimorbidity combinations, except cognitive impairment.

